# Coronary Stent Healing in Cancer Patients—An Optical Coherence Tomography Perspective

**DOI:** 10.3389/fcvm.2021.665303

**Published:** 2021-06-07

**Authors:** Moez Karim Aziz, Joerg Herrmann, Konstantinos Marmagkiolis, Dinu Valentin Balanescu, Teodora Donisan, Bala Pushparaji, Heather Y. Lin, Gerryross Tomakin, Taylor Hoyt, Martin Pham, Jouke Dijkstra, Mehmet Cilingiroglu, Juan Lopez-Mattei, Vlad Zaha, H. Vernon Anderson, Marc D. Feldman, Donald A. Molony, Cezar A. Iliescu

**Affiliations:** ^1^Department of Cardiology, The University of Texas MD Anderson Cancer Center, Houston, TX, United States; ^2^McGovern Medical School at The University of Texas Health Science Center at Houston, Houston, TX, United States; ^3^Department of Cardiovascular Diseases, Mayo Clinic, Rochester, MN, United States; ^4^Department of Internal Medicine, Beaumont Hospital, Royal Oak, MI, United States; ^5^Department of Biostatistics, The University of Texas MD Anderson Cancer Center, Houston, TX, United States; ^6^Division of Cardiology, The University of Texas Health Science Center at San Antonio, San Antonio, TX, United States; ^7^Department of Radiology, Leiden University Medical Center, Leiden, Netherlands; ^8^Department of Internal Medicine, The University of Texas Southwestern Medical Center, Dallas, TX, United States

**Keywords:** stent healing, cardio-oncology, dual antiplatelet therapy discontinuation, acute coronary syndrome, optical coherence tomography

## Abstract

**Objective:** This study assessed stent healing patterns and cardiovascular outcomes by optical coherence tomography (OCT) in cancer patients after drug-eluting stent (DES) placement.

**Background:** Cancer treatment, owing to its cytotoxic and antiproliferative effects, could delay stent healing and increase stent thrombosis risk, especially when dual antiplatelet therapy (DAPT) is discontinued early for oncological treatment. OCT can assess stent endothelialization and other healing parameters, which may provide clinical guidance in these challenging scenarios.

**Methods:** This single-center retrospective study enrolled all cancer patients who underwent OCT for assessment of vascular healing patterns after prior DES placement from November 2009 to November 2018. Primary study endpoints were stent healing parameters, including stent coverage, apposition, degree of expansion, neointimal hyperplasia heterogeneity, in-stent restenosis, stent thrombosis, and overall survival (OS).

**Results:** A total of 67 patients were included in this study. Mean time between DES placement and OCT evaluation was 154 ± 82 days. Stent healing matched published values for DES in non-cancer patients (*P* ≥ 0.063). At 1 year, the OS was 86% (95% confidence interval [CI]: 78–96%) with 0% incidence of acute coronary syndrome. Advanced cancers and active chemotherapies were associated with inferior OS (*P* = 0.024, hazard ratio [HR]: 3.50, 95% CI: 1.18–10.42 and *P* = 0.026, HR: 2.65, 95% CI: 1.13–6.22, respectively), while stent healing parameters were unassociated with OS. Forty-one patients (61%) had DAPT duration ≤6 months.

**Conclusions:** Stent healing of contemporary DES appears similar in cancer and non-cancer patients. Cardiovascular risk of cancer patients after DES placement can be managed to facilitate timely cancer therapies, as the underlying malignancy and active chemotherapy ultimately determine survival.

## Introduction

Approximately 30% of patients with cardiovascular disease have a current cancer diagnosis with 10% of percutaneous coronary interventions (PCI) occurring in cancer patients (1, 2). Thrombocytopenia and bleeding risk related to malignancies or their treatment as well as the need for timely surgical interventions may require premature dual antiplatelet therapy (DAPT) discontinuation, specifically P_2_Y_12_ inhibitors, more often in this patient population. However, discontinuing DAPT prematurely can increase stent thrombotic risk in an already prothrombotic cancer patient population. These competing concerns present a challenging dilemma of when to discontinue DAPT in cancer patients with concomitant coronary artery disease.

Optical coherence tomography (OCT) has been used to guide DAPT discontinuation decisions in cancer patients (3) by offering high resolution and detailed visualization of stented coronary artery segments (4), restenosis, and other stent healing parameters (5–7). Therefore, the current study utilized OCT to accomplish its objectives. The objectives of this study were to evaluate stent healing in cancer patients with previous PCI and drug-eluting stent (DES) implantation, decipher whether stent healing differed from patients without cancer based on published data, assess the impact of cancer stage and active chemotherapy on stent healing, and evaluate the impact of early (<6 months) DAPT discontinuation on overall survival (OS).

## Materials and Methods

### Study Design and Patient Selection

We conducted a single-center, retrospective study of patients with a cancer diagnosis treated at The University of Texas MD Anderson Cancer Center in Houston, Texas, who received coronary stents placed between November 2009 through November 2018. Patients who were treated with PCI with DES implantation, received DAPT, and subsequently underwent OCT evaluation for clinical indications were eligible for inclusion. Clinical indications included abbreviated DAPT course, shortness of breath, acute coronary syndrome, cardiomyopathy, positive biomarkers indicating cancer therapy causing myocarditis, non-specific troponin elevation, and abnormal ECG. OCT at the time of DES implantation was not performed. The local institutional review board approved the study protocol (“A Retrospective Review of Cardiac Catheterization Data in a Cancer Population”); no informed consent was required due to the study's retrospective nature.

Patients' baseline demographics and clinical data were recorded at the time of cardiac catheterization: age, sex, BMI, cardiovascular risk factors (hypertension, smoking history, dyslipidemia, diabetes mellitus, coronary artery disease, and peripheral artery disease), and clinical history including stent number and territory, as well as laboratory data with complete blood counts, creatinine levels, and fasting lipid panel results (8–10).

The antiplatelet regimen was individualized by the operators based on OCT images and evaluation by the cardio-oncology team. Antiplatelet medications were recorded throughout the cancer treatment. Decisions concerning DAPT discontinuation were made based on available literature (3). Patients with a history of mediastinal radiation therapy were excluded to avoid the possible confounding factor of radiation-induced heart disease. Since most PCIs occurred in outside hospital facilities, patients with an unknown stent brand or type, stent placement with multiple stent brands, and undocumented date of stent placement were excluded.

### Stratification of Cancer Diagnosis

Cancers were stratified into early and advanced-stage based on staging guidelines and literature-documented risk factors associated with poor prognosis. Overall, advanced cancer was defined as the presence of metastasis, stage III or higher in solid tumors, relapsed and/or refractory disease, or history of stem cell transplant in hematological malignancies. All cancers where treatment was not with curative intent were considered palliative. When all treatments were exhausted and no active treatment was provided, patients were considered hospice. All patients included in this study had at least 50% or greater probability of a 1-year survival. Sources for this literature survey are provided in the Supplementary Material.

### Aims and Outcomes of the Study

The primary endpoints of the study were stent healing parameters as determined by completeness of strut coverage (11) and apposition (12), degree of expansion (13), neointimal hyperplasia heterogeneity (14), in-stent restenosis (15), stent thrombosis (16), and OS. All parameters recorded have been demonstrated to correlate with OS or with other stent healing parameters (11–16). The 12-month incidence of acute coronary syndrome (ACS) was also recorded. Mean neointimal hyperplasia was also calculated as a secondary assessment of strut and stent coverage. Outcomes were compared to values reported in the literature for populations with cardiovascular disease but without a cancer diagnosis (17–22).

### OCT Analysis

**Figure 1:**
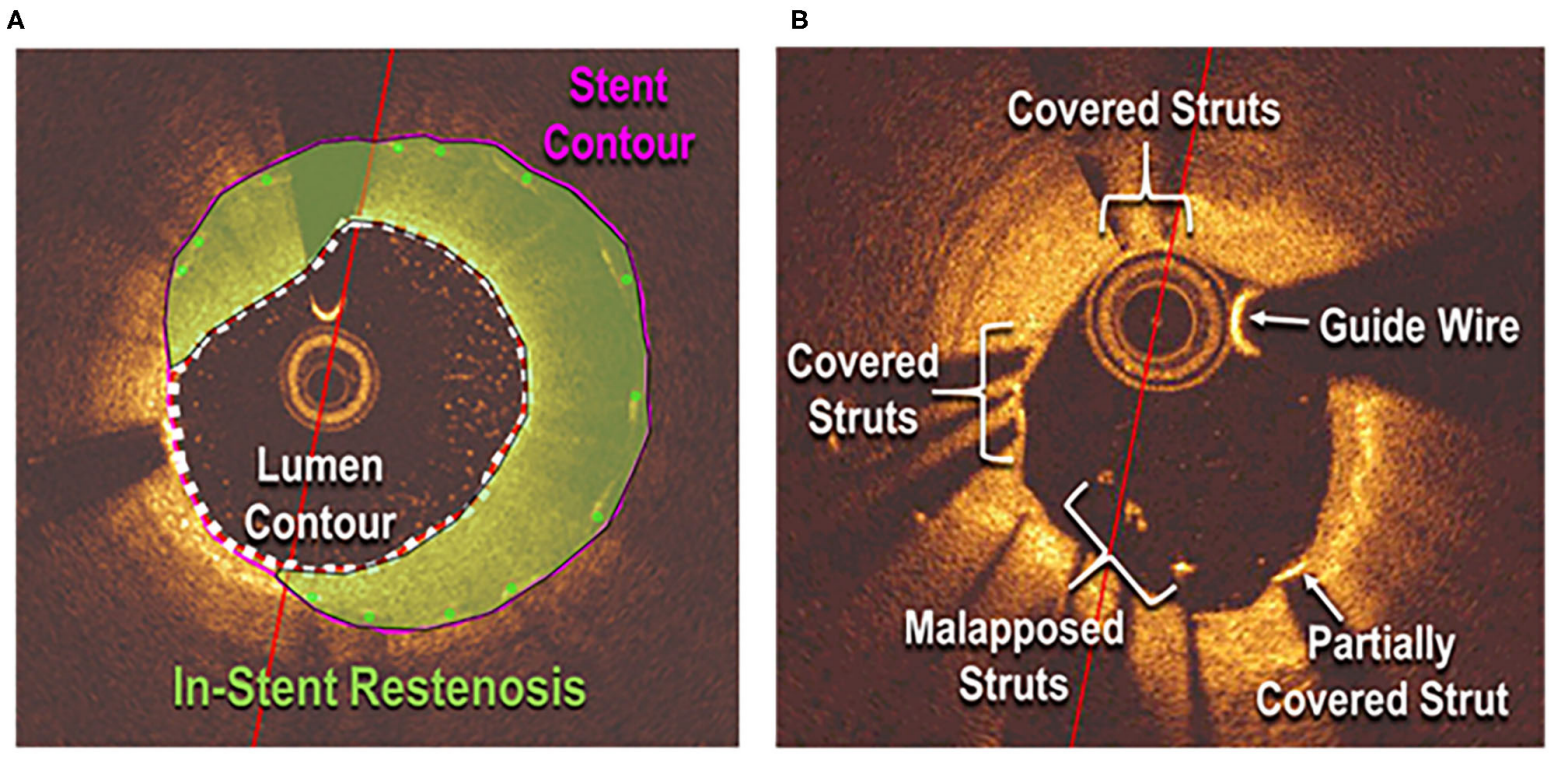
Sample assessment of stent healing parameters. Stent **(A)** and strut **(B)** measurements.

A C7 Dragonfly OCT catheter and C7-XR OCT intravascular imaging system (St. Jude Medical, St. Paul, MN) were used to obtain OCT data (3). OCT images were analyzed in a semi-automated fashion using the proprietary software QCU-CMS, developed by Dijkstra et al. (Leiden University Medical Center) (23). Manual corrections for detection errors were performed by two independent observers (M.K.A. and C.A.I.). Strut apposition and coverage were detected by whether the strut was located above, at, or below the lumen contour (Figure 1). Data were excluded from analysis if during pullback adequate blood clearance was not obtained or stent struts were not clearly identified. Follow-up was obtained through review of hospital and clinic records.

### Statistical Analysis

OS was defined as the time from OCT measurement to death or last contact and was estimated by the Kaplan-Meier method. The log-rank test was used to analyze differences in OS between patients with early-stage and advanced cancer diagnoses. Parameters affecting OS were established with Cox regression. An ANOVA variance analysis with a linear mixed-effect model was used to assess the relationship between stent brand, patient demographics, clinical characteristics, and stent healing parameters to account for patients with multiple stents. The Wilcoxon signed rank test was used to compare stent measurements with corresponding published values in patients without a cancer diagnosis. Studies from which published values were derived are cited in the manuscript. Comparisons were made only if the number of days from DES placement to OCT fell within the time range from which the published value was derived to ensure validity. A 2-sided *P* < 0.05 was considered statistically significant. SAS version 9.4 and S-Plus version 8.04 were used to carry out the computations for all analyses.

## Results

### Study Population

**Figure 2:**
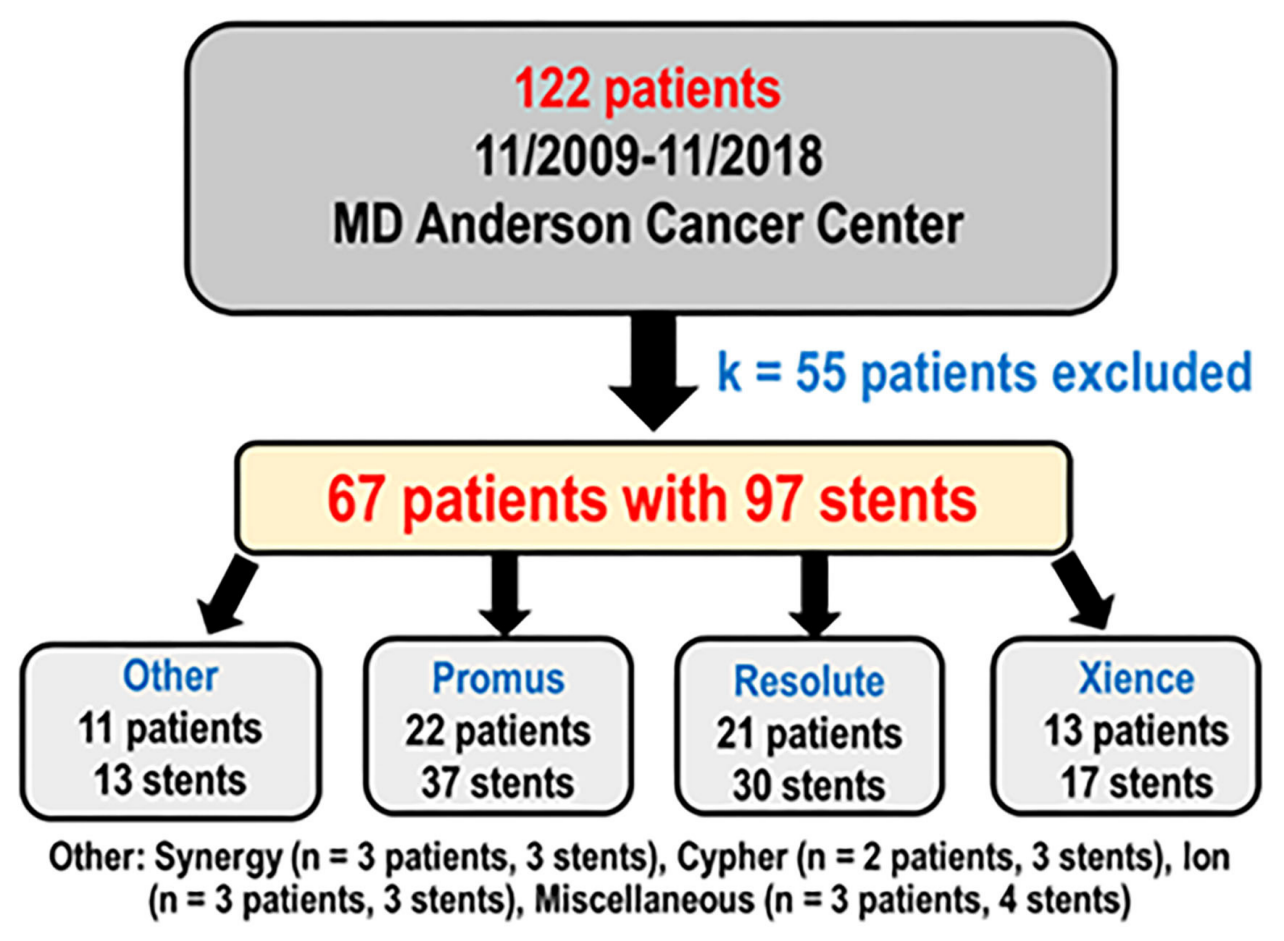
Study design and stent brand distribution.

**Figure 3:**
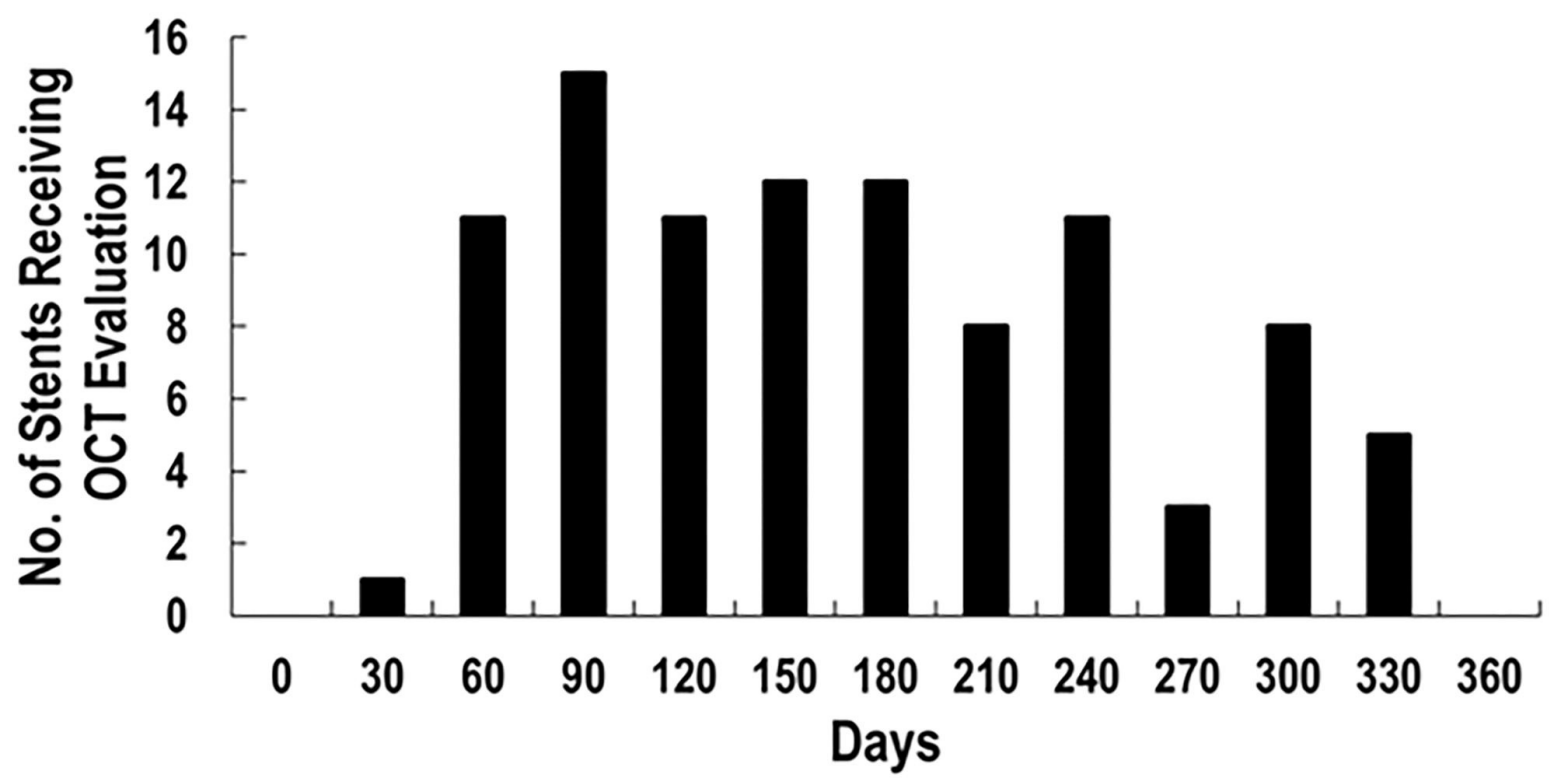
Distribution of days between stent placement and OCT evaluation.

One hundred twenty-two patients had coronary stents placed from November 2009 through November 2018 and underwent OCT as part of their clinical care. After 55 patients with incomplete data were excluded, there were 67 patients with 97 stents analyzed (Figure 2) with more than 15,000 strut cross sections. Baseline demographics are presented in Table 1. Patients were predominantly male (82.09%) with high prevalence of cardiovascular risk factors: hypertension (91.04%), smoking (58.21%), dyslipidemia (94.03%), diabetes (34.33%), and family history of coronary artery disease (40.30%). Thirteen of these patients (19.40%) were undergoing active chemotherapy; 8 of these 13 patients had history of chemotherapy (11.94%). The mean time between stent placement and OCT evaluation was 154 ± 82 days (Figure 3). Forty-nine of 67 patients (73%) underwent OCT to evaluate the possibility of an abbreviated DAPT course. Forty-one of 67 patients (61%) [with 59 of 97 stents (61%)] had DAPT discontinued for cancer treatment <6 months after stent placement. The mean time between stent implantation and DAPT discontinuation for this subset was 105 ± 45 days.

**Table 1:** Descriptive data for the study cohort (*N* = 67).

**Covariates**	**Mean ± standard deviation, median (Min, Max), or No. (valid %)**
**Demographics**	
Age, y	65.72 ± 9.04, 66 (41, 84)
Body mass index, kg/m^2^	29.9 ± 6.22 28.7 (20.35, 48.78)
Men	55 (82.09)
Women	12 (17.91)
**Cardiovascular risk factors**	
Smoking	39 (58.21)
Hypertension	61 (91.04)
Systolic blood pressure, mmHg	135.17 ± 21.3, 132 (92, 198)
Diastolic blood pressure, mmHg	73.08 ± 11.25, 72 (49, 102)
Dyslipidemia	63 (94.03)
Diabetes	23 (34.33)
Family history of coronary artery disease	27 (40.30)
**Clinical history**	
Heart failure	12 (17.91)
Ejection fraction, %	55.91 ± 10.09, 57.5 (19, 70)
Coronary artery disease	64 (95.52)
Myocardial infarction	26 (38.81)
Coronary artery bypass graft	8 (11.94)
Previous number of stents	
1	44 (65.67)
2	15 (22.39)
3	8 (11.94)
Peripheral artery disease	13 (19.40)
Chronic renal insufficiency	12 (17.91)
**Indications for OCT analysis**	
Abbreviated DAPT course	49 (73.13)
Shortness of breath	12 (17.91)
Acute coronary syndrome	11 (16.42)
Cardiomyopathy	2 (2.99)
Positive biomarkers of cancer therapy causing myocarditis	1 (1.49)
Non-specific troponin elevation	1 (1.49)
Abnormal ECG	5 (7.46)
**Cancer data**	
Solid	57 (85.07)
Hematologic	10 (14.93)
Advanced	40 (59.70)
Chemotherapy	13 (19.40)
Active	13 (19.40)
History of chemotherapy	8 (11.90)
**Laboratory data**	
Absolute neutrophil count, cells/μL	4.66 ± 2.3, 4.16 (0, 15.59)
Hemoglobin, g/dL	12.83 ± 1.77, 12.9 (9.6, 17.4)
Platelet count, × 10^3^/μL	212.58 ± 82.67, 201.5 (9, 439)
Creatinine pre-OCT, mg/dL	1.23 ± 1.19, 0.98 (0.48, 8.68)
Creatinine post-OCT, mg/dL	1.21 ± 0.98, 0.99 (0.57, 7.42)
Triglycerides, mg/dL	130.55 ± 49.64, 122 (47, 236)
Total cholesterol, mg/dL	145.66 ± 39.31, 138.5 (91, 239)
High-density lipoprotein, mg/dL	44.02 ± 13.64, 40 (26, 76)
Low-density lipoprotein, mg/dL	79.51 ± 36.71, 71 (36, 192)
DAPT characteristics^*^	
Remained on aspirin	59 (88.06)
Remained on P2Y12 inhibitor: Remained on clopidogrel	2 (2.99)
Remained on ticagrelor	2 (2.99)
Complete discontinuation	5 (7.46)
Single antiplatelet treatment	55 (82.09)
Dual antiplatelet treatment	4 (5.97)
Not recorded	3 (4.48)
**Subsequent events**	
Acute coronary syndrome	0.00 (0.00)
Death	25 (37.31)

*DAPT, dual antiplatelet therapy; OCT, optical coherence tomography*.

* *DAPT discontinuation occurred at <6 mo post-placement in 41 out of 67 patients (61%)*.

### Strut and Stent Parameters

**Figure 4:**
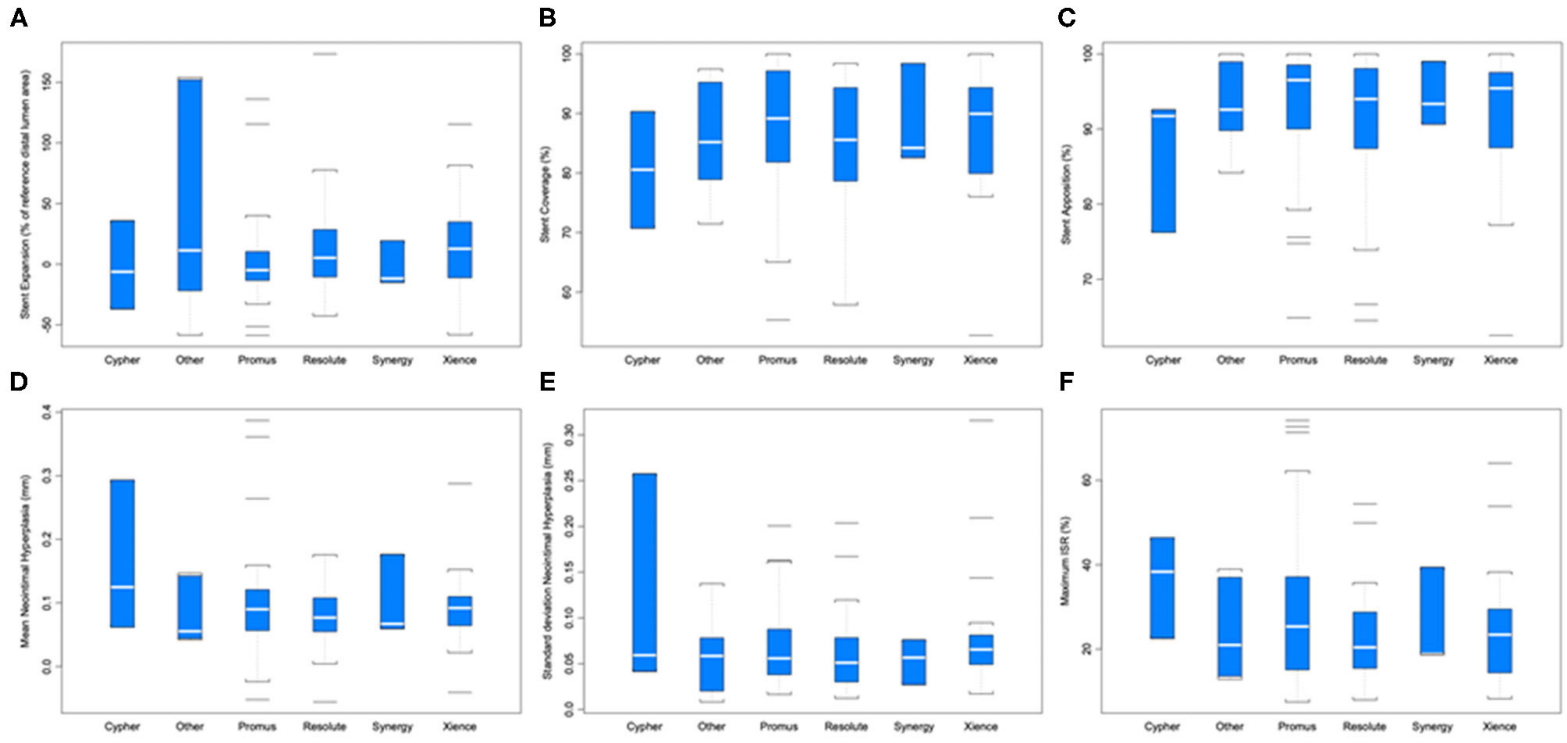
Stent healing parameters by different stent brands. **(A)** Stent expansion (*P* = 0.502). **(B)** Stent coverage (*P* = 0.707). **(C)** Stent apposition (*P* = 0.210). **(D)** Mean neointimal hyperplasia (mm) (*P* = 0.736). **(E)** Standard deviation of neointimal hyperplasia (*P* = 0.591). **(F)** Maximum in-stent restenosis (ISR; *P* = 0.204).

Strut coverage, completeness of apposition, and degree of expansion, as well as neointimal hyperplasia and maximum in-stent restenosis are reported in Figure 4. ANOVA with linear mixed-effect model demonstrated equivalent stent healing among stent brands (*P* ≥ 0.204). Cancer prognosis was not associated with stent healing (early vs. advanced; *P* ≥ 0.095). Active chemotherapy and history of chemotherapy did not impact stent healing (*P* ≥ 0.194); chemotherapies in this patient population included cisplatin, docetaxel, FOLFIRINOX regimen, carboplatin, pembrolizumab, pemetrexed, MVAC regimen, cabazitaxel, melphalan, R-CHOP regimen, ibrutinib, cytarabine, and bevacizumab.

The impact of baseline characteristics and their association with stent healing are presented in Table 2. Stratified comparisons to literature values by follow-up duration of OCT after DES placement and stent brand (Resolute, Promus, and Xience stents) were performed with results noted in Table 3 (17–22). For inclusion in a particular comparison, the follow-up duration of OCT needed to fall within the follow-up duration of stents included in the published study that generated a literature value. Stent healing in our cohort of cancer patients was similar to published data from patients with only cardiovascular disease (*P* ≥ 0.063).

**Table 2:** Descriptive OCT stent measurements (*N* = 97 stents in 67 patients).

**Covariates**	**Strut coverage, %**	**Strut apposition, %**	**Strut expansion, %**	**Mean neointimal hyperplasia, mm**	**Neointimal hyperplasia heterogeneity, mm**	**In-stent restenosis, %**
**Demographics**						
Men	85.45 ± 11.78	91.45 ± 9.3	5.13 ± 36.54	0.1 ± 0.06	0.07 ± 0.05	12.03 ± 9.53
**Cardiovascular risk factors**						
Smoking	86.45 ± 9.88	93.17 ± 7.16	7.56 ± 38.57	0.09 ± 0.04	0.06 ± 0.04^*^	11.45 ± 5.14
Hypertension	85.96 ± 11.12	91.68 ± 8.95	8.91 ± 41.9	0.1 ± 0.07	0.07 ± 0.06^*^	12.22 ± 9.28
Dyslipidemia	85.71 ± 11.28	91.71 ± 8.93	10.33 ± 43.28	0.09 ± 0.07	0.07 ± 0.05	12.15 ± 9.16
Diabetes	87.03 ± 9.72	92.37 ± 8.27	−3.05 ± 31.71^*^	0.1 ± 0.06	0.07 ± 0.05	12.76 ± 6.83
Family history of coronary artery disease	85.16 ± 11.89	91.24 ± 9.11	8.92 ± 43.02	0.1 ± 0.09	0.07 ± 0.06	13.6 ± 10.79
**History**						
Heart failure	86.54 ± 11.57	92.25 ± 7.56	1.06 ± 44.49	0.11 ± 0.06	0.07 ± 0.04	15.05 ± 9.07
Coronary artery disease	85.95 ± 11.16	92.04 ± 8.79	10.1 ± 43.72	0.09 ± 0.07	0.07 ± 0.05^*^	12.03 ± 9.04
Myocardial infarction	87.09 ± 9.51	93.37 ± 6.16	10.3 ± 44.34	0.11 ± 0.09	0.08 ± 0.06	14.11 ± 10.06
Coronary artery bypass graft	80.31 ± 10.66	89.39 ± 9.07	21.09 ± 50.62	0.06 ± 0.06	0.06 ± 0.03	8.22 ± 8.2
Indications for OCT analysis^**^						
Abbreviated DAPT course	85.21 ± 11.45	91.22 ± 9.31	12.88 ± 45.98	0.09 ± 0.07	0.07 ± 0.04	11.54 ± 9.39
Shortness of breath	89.89 ± 9.51	94.77 ± 6.72	0.57 ± 43.65	0.12 ± 0.07	0.07 ± 0.06	14.70 ± 8.64
Acute coronary syndrome	82.61 ± 9.76	90.46 ± 6.62	10.43 ± 44.39	0.10 ± 0.07	0.09 ± 0.08	11.95 ± 6.18
Cardiomyopathy	81.81 ± 11.47	85.59 ± 9.42	−5.22 ± 7.46^*^	0.12 ± 0.06	0.09 ± 0.04	16.37 ± 9.17
Abnormal ECG	93.50 ± 4.75^*^	97.19 ± 2.20^*^	−1.71 ± 23.11	0.12 ± 0.08	0.07 ± 0.06	16.66 ± 12.30
**Cancer data**						
History of chemotherapy	81.52 ± 13.56	89.70 ± 10.26	11.09 ± 56.38	0.08 ± 0.08	0.07 ± 0.05	10.30 ± 12.46
Active chemotherapy	84.24 ± 13.8	90.29 ± 10.66	7.29 ± 47.64	0.09 ± 0.08	0.08 ± 0.06	12.55 ± 11.69
Advanced	86.77 ± 10.99	92.55 ± 8.4	9.4 ± 44.16	0.1 ± 0.08	0.07 ± 0.06	13.31 ± 10.03
**Antiplatelet medications**						
Remained on aspirin	85.8 ± 11.33	91.53 ± 9.1^*^	11.15 ± 46.04	0.1 ± 0.08	0.07 ± 0.06^*^	12.19 ± 9.52
Remained on clopidogrel	87.35 ± 1.96	91.53 ± 1.36	5.92 ± 25.31	0.14 ± 0.01^*^	0.09 ± 0.03	18.7 ± 5.13^*^
Remained on ticagrelor	86.51 ± 11.22	93.34 ± 5.73	45.27 ± 45.93	0.1 ± 0.08	0.07 ± 0.02	11.01 ± 7.89

* *P < 0.05, used to determine association with stent parameter*.

** *Non-specific troponin elevation and positive biomarkers indicating cancer therapy causing myocarditis were not included in this analysis due to small sample sizes of only 1 patient for each of these groups*.

**Table 3:** Stent healing comparison with literature values.

**Stent parameter**	**Promus (****17****)**				**Resolute (****18****–****20****)**				**Xience (****17**, **21**, **22****)**			
	**Mean ± SD cancer patients**	**Mean ± SD literature**	***P***	**Time (days)**	**Mean ± SD cancer patients**	**Mean ± SD literature**	***P***	**Time (days)**	**Mean ± SD cancer patients**	**Mean ± SD literature**	***P***	**Time (days)**
Stent coverage (%)	89.49 ± 12.38	97 ± 7	0.313	241–360	79.09 ± 15.61	95.5 ± 5.5	0.063	31–60	82.15 ± 3.15	73.3 ± 21.3	0.5	31–60
Stent apposition (%)	96.28 ± 5.26	99.8 ± 0.8	0.125	241–360	87.81 ± 12.94	98.1 ± 1.9	0.125	31–60	99.24 ± 0.47	98.7 ± 2.8	0.5	241–360
Mean neointimal hyperplasia (mm)	0.13 ± 0.12	0.105 ± 0.082	1.0	241–360	0.06 ± 0.08	0.07 ± 0.01	1.0	31–60	0.13 ± 0.04	0.091 ± 0.08	0.5	241–360
In-stent restenosis (%)	NA	NA	NA	NA	NA	NA	NA	NA	33.37 ± 6.29	36.8 ± 15.6	0.625	91–180

### Clinical Outcomes

**Figure 5:**
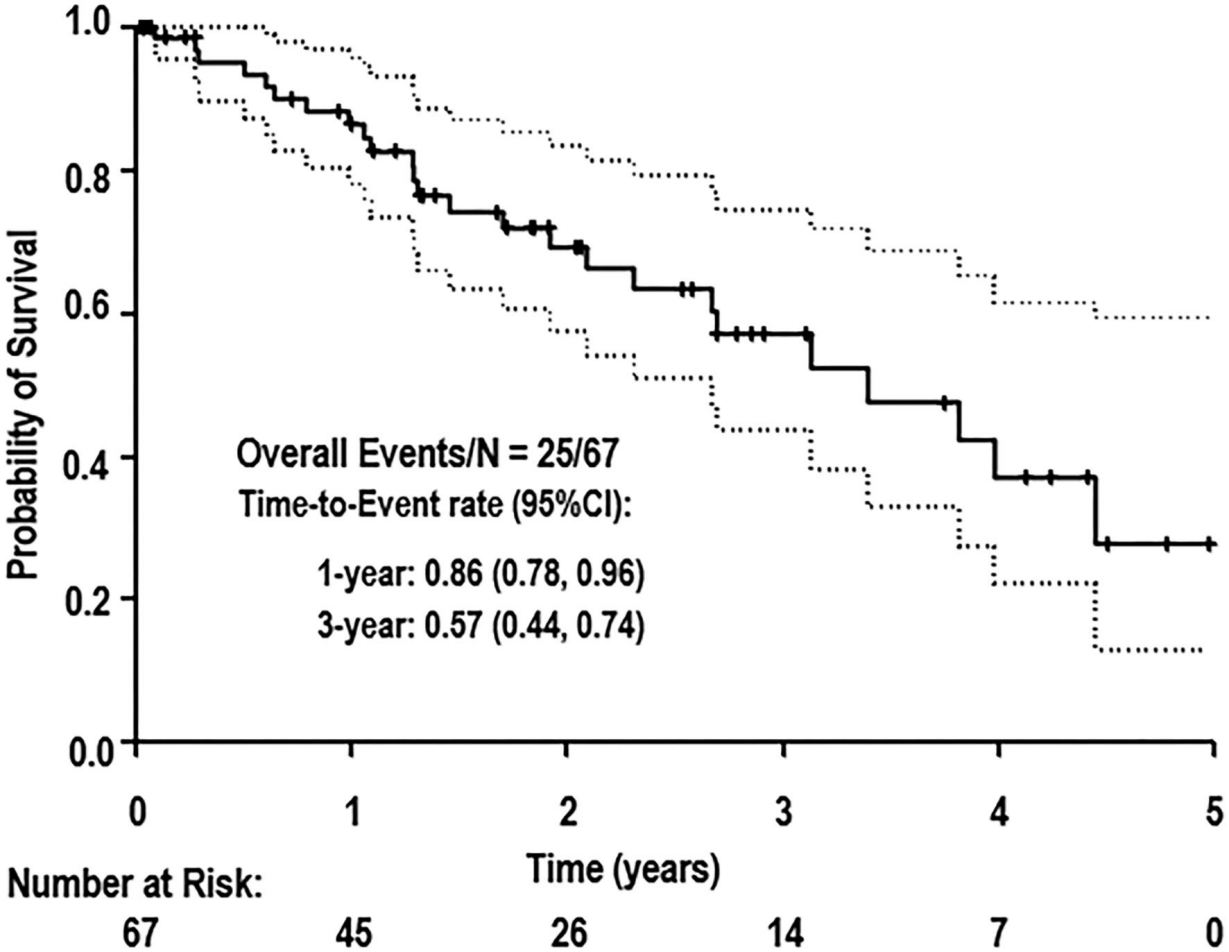
Overall survival of studied population. Survival at 1 and 3 years provided.

The median follow-up time was 2.5 years. Median OS was 3.4 years (95% confidence interval [CI]: 2.3–4.5 years). Long-term survival was driven by cancer-related mortality. The OS at 1 year from stent placement was 86% and further decreased to 57% at 3 years (Figure 5). The cause of death for all patients was cancer. Neither stent thrombosis nor ACS occurred in the analyzed cohort of patients. Deep venous thrombosis incidence at 1 year was 11.9% (eight patients).

### Factors Associated With Survival

**Figure 6:**
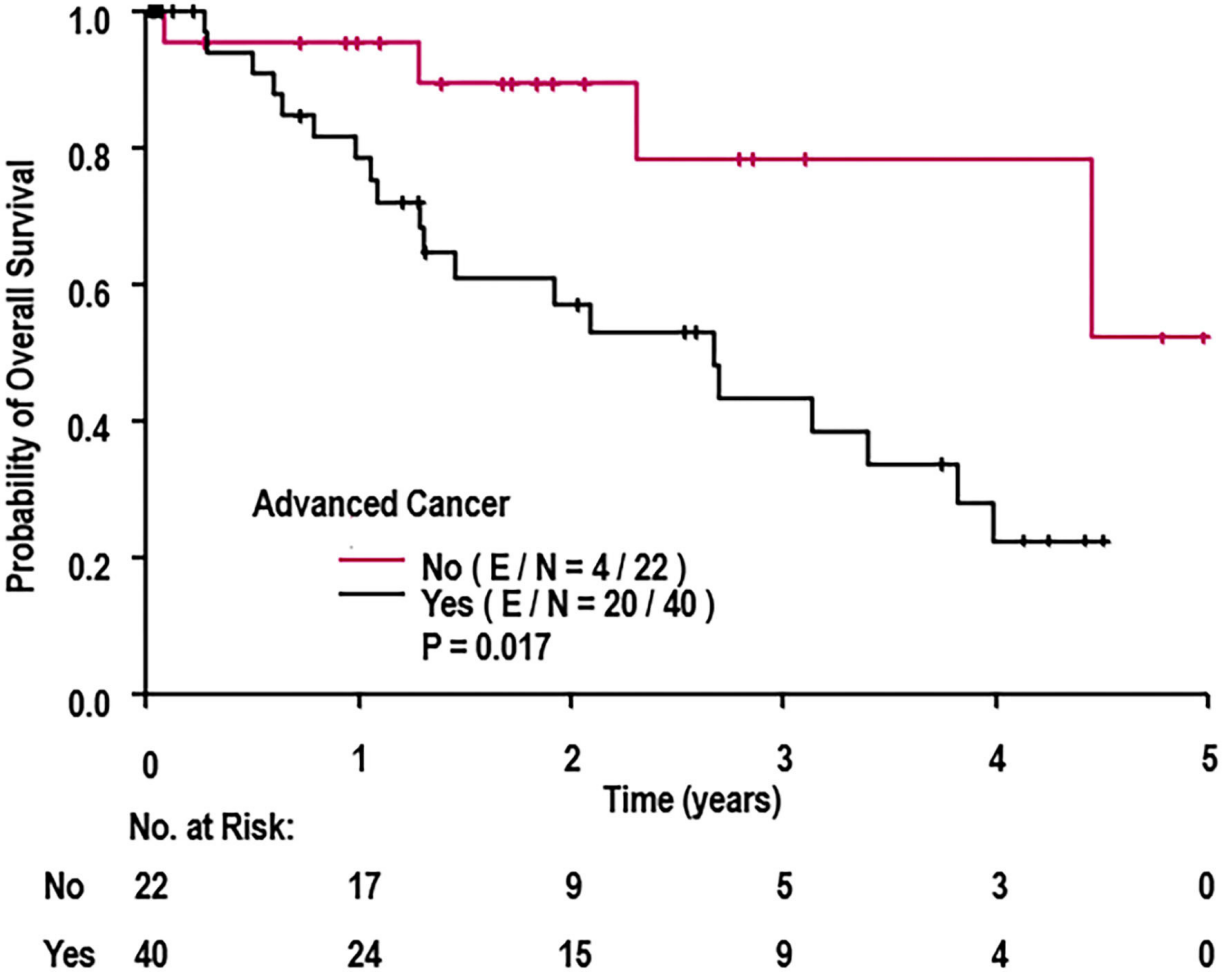
Overall survival of cohort when stratified by cancer prognosis (early vs. advanced). E, events; N, number of patients; OS, overall survival. *P*-value determined from log-rank test.

A univariate Cox regression was conducted to determine which patient characteristics, stent parameters, and strut parameters correlated with OS (Table 4). Of all characteristics noted, only cancer prognosis (early vs. advanced), active chemotherapy, and aspirin discontinuation correlated with OS. Continued use of aspirin was associated with longer OS (hazard ratio [HR]: 0.18, 95% CI: 0.04–0.88, *P* = 0.034). Patients on active chemotherapy had a higher mortality with an HR of 2.65 (95% CI: 1.13–6.22, *P* = 0.026). Patients with an advanced cancer stage had a higher mortality with an HR of 3.50 (95% CI: 1.18–10.42, *P* = 0.024). None of the stent healing parameters, including strut coverage, strut apposition, stent expansion, in-stent restenosis, mean neointimal hyperplasia, and heterogeneity of neointimal hyperplasia, correlated with OS (*P* ≥ 0.098). Differences in OS between early and advanced cancers were significantly different as per the log-rank test (*P* = 0.017; Figure 6).

**Table 4:** Cox proportional Hazard model to determine associations of baseline characteristics and stent healing parameters with survival.

**Parameter**	**Classification Method**	***P***	**Hazard Ratio (95% CI)**
**Demographics**			
Sex	Female vs. Male	0.144	0.12 (0.01–2.08)
Age	Per year increase	0.643	1.01 (0.96–1.06)
**Cardiovascular risk factors**			
Smoking	Yes vs. No	0.836	1.09 (0.49–2.41)
Hypertension	Yes vs. No	0.902	1.14 (0.15–8.70)
Dyslipidemia	Yes vs. No	0.792	0.76 (0.10–5.85)
Diabetes	Yes vs. No	0.348	1.48 (0.65–3.35)
Family history of coronary artery disease	Yes vs. No	0.828	0.91 (0.40–2.07)
**History**			
Heart failure	Yes vs. No	0.099	2.11 (0.87–5.11)
Coronary artery disease	Yes vs. No	0.437	2.22 (0.30–16.59)
Myocardial infarction	Yes vs. No	0.092	0.43 (0.16–1.15)
Coronary artery bypass graft	Yes vs. No	0.694	1.28 (0.38–4.31)
Peripheral artery disease	Yes vs. No	0.613	0.78 (0.29–2.08)
Chronic renal insufficiency	Yes vs. No	0.428	1.51 (0.55–4.13)
Indications for OCT analysis^**^			
Abbreviated DAPT course	Yes vs. No	0.110	0.48 (0.20–1.18)
Shortness of breath	Yes vs. No	0.054	2.44 (0.98–6.07)
Acute coronary syndrome	Yes vs. No	0.319	1.76 (0.58–5.38)
Cardiomyopathy	Yes vs. No	0.237	2.42 (0.56–10.46)
Abnormal ECG	Yes vs. No	0.499	1.67 (0.38–7.43)
**Cancer Data**			
History of chemotherapy	Yes vs. No	0.072	2.72 (0.92–8.08)
Active chemotherapy	Yes vs. No	0.026^*^	2.65 (1.13–6.22)
Advanced (cancer types in Supplemental Material)	Advanced vs. early–stage	0.024^*^	3.50 (1.18–10.42)
**Antiplatelet medications**			
Remained on aspirin	Yes vs. No	0.034^*^	0.18 (0.04–0.88)
Remained on clopidogrel	Yes vs. No	0.353	2.62 (0.34–20.12)
Remained on ticagrelor	Yes vs. No	0.658	0.50 (0.02–10.89)
**Stent healing parameters**			
Maximum in-stent restenosis, %	Per unit increase	0.720	1.00 (0.97–1.02)
Mean neointimal hyperplasia, mm	Per unit increase	0.400	0.08 (0.00–28.95)
*^†^*log_2_(Neointimal hyperplasia heterogeneity, mm)	Per fold increase	0.651	0.91 (0.61–1.36)
Mean strut expansion, %	Per unit increase	0.125	0.99 (0.98–1.00)
Mean strut coverage, %	Per unit increase	0.119	0.98 (0.95–1.01)
Mean strut apposition, %	Per unit increase	0.098	0.97 (0.93–1.01)

* *P < 0.05, used to determine association with overall survival, NR, not reached*.

** *Non-specific troponin elevation and positive biomarkers indicating cancer therapy causing myocarditis were not included in this analysis due to small sample sizes of only 1 patient for each of these groups*.

† *Log_2_ transformation of the original variables required due to right-skewed distribution*.

## Discussion

While past studies have used parameters associated with stent healing to guide DAPT discontinuation in cancer patients (3), this is the first study to compare stent healing in cancer patients who underwent intravascular imaging with OCT after DES implantation to a non-cancer population. With the increased incidence of patients with concomitant cardiovascular disease and cancer due to shared risk factors and population aging, the question of how to manage patients with PCI has increased relevance; stent healing is an important part of this question.

Past literature based on animal and *in-vitro* human cell and tissue studies has generated an expectation of delayed stent healing in cancer patients. Tissue factor (24), von Willebrand factor (25), and ADP (26) have been deemed common metabolites contributing to stent thrombosis and cancer pathogenesis; therefore, one would naturally expect that heightened levels of these metabolites from cancer pathogenesis would delay stent healing by contributing to stent thrombosis. However, the accelerated healing kinetics of contemporary DES (27) appear unaffected by vascular toxicities of cancer therapies and biological deterioration from cancer progression. The time scale of stent healing for contemporary DES has shortened to the extent that stent healing is now minimally impacted by cancer pathogenesis. When zooming in on the stent healing process, the rather short time interval required for healing for DES appears very close to a biological plateau minimally impacted by cancer or its treatments.

OCT evaluation is a valuable and effective tool to analyze stent healing and drives a convergence and a quantitative approach where unique clinical characteristics and treatments would make any form of randomization impractical. In addition to the numerous patient characteristics that affect stent thrombosis and in-stent restenosis including age (28), coronary artery disease (29), lack of appropriate statin use (30), low high-density lipoprotein (31), plasma-oxidized low-density lipoprotein (32), diabetes mellitus (33), renal failure (34), prior myocardial infarction (35), prior PCI (35), family history of cardiovascular disease (36), and low ejection fraction (37), increased additional complexity is brought by stent characteristics: polymer, platform, and eluting medication; (38) operator variability; (39) and the prothrombotic nature of the malignancy (40). From 2009 to 2011, a study by Shafiq et al. indicated a 69% variation in the likelihood of DES implantation among physicians in similar hospital settings caring for patients with identical characteristics (39). Since P_2_Y_12_ inhibitor discontinuation decisions in the cancer population rely on stent healing parameters (3), a study based purely on clinical characteristics to address the risk of P_2_Y_12_ inhibitor discontinuation also represents an impossible task.

A randomized control trial of 117,762 patients conducted in 2012 indicated differences in restenosis and thrombosis between stent brands (41). While assessment of the impact of stent brand in thrombosis incidence is difficult due to the small sample size and absent events, all stent healing parameters trended similarly regardless of stent brand. Of note, in-stent restenosis was similar among brands. Despite differences in platform, polymer, and eluting medication, overall advancements in stent technology may have abated these once clinically significant differences (42).

Since advances in stent technology have reduced the time scale of stent healing, the finding that cancer no longer or minimally impacts stent healing in newer-generation DES is increasingly plausible. Of note, stent healing was also unassociated with active chemotherapy. Ultimately, these findings can generate optimism and increase involvement to address cardiovascular comorbidities and improve resilience to cancer treatment challenges by permitting cancer pathogenesis and stent healing to be treated as two independent processes. Supporting this notion is the non-negligible incidence of deep venous thrombosis consistent with malignancy-based hypercoagulability despite routine prophylaxis and zero stent thrombosis.

This idea elicits the question of whether patients may receive cancer treatment independent of stent healing by discontinuing the P_2_Y_12_ inhibitor to manage bleeding risk. Our OCT study demonstrated the relative safety of premature P_2_Y_12_ discontinuation independent of cancer stage or treatment. Zero ACS events occurred at 1 year, including no stent thrombosis despite more than half of this patient population discontinuing DAPT at <6 months and the prothrombotic nature of cancer and cancer treatments (43). These results observed for patients with cancer are similar to non-cancer patients (44). A recent randomized control trial that included both cancer and non-cancer patients with indications for remaining on DAPT for only 1 month is also consistent with these results (45). In our study, neither P_2_Y_12_ inhibitor discontinuation decisions themselves nor the stent healing parameters used to generate these decisions impacted OS. Therefore, cancer status and active chemotherapy, due to their association with OS, should be prioritized when evaluating risks associated with P_2_Y_12_ inhibitor discontinuation. Emergent cancer treatments should not be delayed merely due to DAPT discontinuation guidelines.

One may ask why not continue the traditional practice of stenting with bare metal stents (BMS) in cancer patients to circumvent the question of premature P_2_Y_12_ discontinuation? While BMS provide rapid endothelization, shorter DAPT duration, and relatively low stent thrombosis risk compared to the first-generation of DES (46), second- and third-generation DES have demonstrated even lower stent thrombosis risk than BMS (47). With a contemporary almost default stenting with DES, we have witnessed an accelerated decrease of DAPT duration over the last 5 years as stent designs have improved. While European Society of Cardiology (ESC) guidelines permit 1-month DAPT with DES for specific indications, current American Heart Association (AHA)/American College of Cardiology (ACC) guidelines still indicate that DAPT can be shortened to 3–6 months in patients with increased bleeding risk (48). However, in our cohort of cancer patients, more than half the patients discontinued DAPT <6 months (mean duration 3–4 months) after stent placement. Examination of the two most commonly used P_2_Y_12_ inhibitors, clopidogrel and ticagrelor, indicates that their discontinuation had no effect on survival. While continuation of aspirin was associated with strut apposition and appeared to improve OS, it may have also been associated with cancer prognosis (early vs. advanced). While recognizing that patients with greater cardiovascular risk benefit from longer DAPT (>12 months) (49), each cancer patient with cardiovascular burden should have a personalized approach to DAPT discontinuation that accounts for cancer status and prognosis.

### Limitations

A major limitation of this study is the lack of a control group when comparing stent healing of cancer patients; the center at which this study was conducted treats only patients with a cancer diagnosis. Therefore, a sample cohort with purely cardiovascular pathologies who could be directly compared to the studied population under identical conditions could not be constructed. Ultimately, populations from various published studies were used as comparison groups.

Another limitation concerns the stents used in this study. An ideal scenario for a cancer patient who requires PCI would include 4 or preferably 2 weeks of DAPT, with overall low or absent thrombotic risk and minimal in-stent restenosis during a proinflammatory and prothrombotic treatment. Select stents are approaching these goals; however, they are too recent to be included in this study (45).

Ideally, immediate status of stent healing and placement can serve as an important indicator of late stent healing status. However, since the center at which this study was conducted is a tertiary care center, DES implantation occurred at outside hospitals in which OCT was not conducted immediately after stent implantation. Therefore, information regarding initial stent status and its relationship to OS in this population is unavailable. Nevertheless, in a study published in the *Journal of American College of Cardiology* in 2020, no difference in survivorship was observed when comparing cancer patients with intravenous ultrasound or OCT taken during DES placement vs. cancer patients receiving OCT follow-up after DES placement (50).

Additionally, while our study addressed the relative safety of early P_2_Y_12_ inhibition discontinuation irrespective of cancer stage and treatment, our time frame was insufficient to address the interesting aspect of prolonged (>1 year) P_2_Y_12_ inhibition and its impact on cancer or cardiovascular mortality (51). One previous study examining prolonged P_2_Y_12_ inhibition in cancer patients suggests that it had no effect on cancer or mortality (51).

While cause of death was appropriately established based on the medical record, the retrospective nature of this study primarily establishes associations; causations of additional or aggregate findings are challenging to validate. Furthermore, the time from stent placement to OCT in the studied population was 154 ± 82 days, implying that these conclusions regarding stent healing can only be applied for healing occurring during this time frame. Future studies should assess stent healing *via* OCT evaluation beyond this limited time frame. Additionally, OCT devices are currently unable to specifically pinpoint fibrin deposition, which would be prothrombotic despite appearing as covered and healed stent struts (52). Finally, published values were not available for all measured parameters of each individual stent brand.

## Conclusions

Cancer patients with coronary artery disease receiving DES appear to have a primarily cancer-driven prognosis; therefore, decisions concerning DAPT and especially P_2_Y_12_ inhibitor discontinuation should prioritize cancer treatment and active chemotherapy considerations over thrombotic risk. The comparable stent healing visualized by OCT between cancer and non-cancer patients regardless of stent brand and the P_2_Y_12_ inhibitor discontinuation not impacting survival should encourage a personalized approach to stent healing management that accounts for cancer status and prognosis. Emergent cancer treatments should be prioritized since cancer status and active chemotherapy ultimately determine OS.

## Data Availability Statement

The raw data supporting the conclusions of this article will be made available by the authors, without undue reservation.

## Ethics Statement

The studies involving human participants were reviewed and approved by MD Anderson Institutional Review Board. Written informed consent for participation was not required for this study in accordance with the national legislation and the institutional requirements.

## Author Contributions

MKA participated in conception and design, analysis, interpretation of data, drafting of the manuscript, and revising it critically. JH, KM, MC, and JL-M participated in conception of study and revising the manuscript critically. DVB, TD, VZ, and HA participated in revising the manuscript critically. BP, HL, GT, and JD participated in analysis and interpretation of data. TH and MF participated in analysis, interpretation of data, and revising the manuscript critically. MP participated in drafting of the manuscript. DM participated in study design and revising the manuscript critically. CI participated in conception and design, analysis, interpretation of data, and revising the manuscript critically. All authors contributed to the article and approved the submitted version.

## Conflict of Interest

The authors declare that the research was conducted in the absence of any commercial or financial relationships that could be construed as a potential conflict of interest.
