# Supplementary material for: Coronary Stent Healing in Cancer Patients—An Optical Coherence Tomography Perspective
Source: Front Cardiovasc Med. 2021 Jun 7;8:665303. doi: 10.3389/fcvm.2021.665303 (PMC8215158; doi:10.3389/fcvm.2021.665303)
Supplement: Supplementary file 1 [file Data_Sheet_1.DOCX]

**Supplementary Material:** Prognostic Models and Criteria for Determining Advanced Cancers

| **Type of Cancer** | **Citation from which Criteria Were Obtained** |
| --- | --- |
| Renal cell cancer | Ko J, Xie W, Kroeger N, et al. The International Metastatic Renal Cell Carcinoma Database Consortium model as a prognostic tool in patients with metastatic renal cell carcinoma previously treated with first-line targeted therapy: a population-based study. *Lancet Oncol*. 2015;16(3):293-300 |
| Prostate cancer | Halabi S, Lin CY, Kelly WK, et al. Updated prognostic model for predicting overall survival in first-line chemotherapy for patients with metastatic castration-resistant prostate cancer. *J Clin Oncol*. 2014;32(7):671–677. |
| Prostate cancer | Heller G, McCormack R, Kheoh T, et al. Circulating tumor cell number as a response measure of prolonged survival for metastatic castration-resistant prostate cancer: a comparison with prostate-specific antigen across five randomized phase III clinical trials. *J Clin Oncol*. 2019;36(6):572-580. |
| Thyroid cancer | Maino F, Forleo R, Pacini F. Prognostic indicators for papillary thyroid carcinoma. *Expert Rev Endocrinol Metab*. 2017;12(2):101-108. |
| Gastric cancer | Siewert JR, Böttcher K, Stein HJ, Roder JD. Relevant prognostic factors in gastric cancer: ten-year results of the German Gastric Cancer Study. *Ann Surg*. 1998;228(4):449–461. |
| Gastric cancer | Nakamuva K, Ueyarna T, Yao T, et al. Pathology and prognosis of gastric carcinoma. Findings in 10,000 patients who underwent primary gastrectomy. *Cancer*. 1992;70(5):1030-1037. |
| Gastric cancer | Adachi Y, Yasuda K, Inomata M, Sato K, Shiraishi N, Kitano S. Pathology and prognosis of gastric carcinoma. *Cancer*. 2000;89(7):1418-1424. |
| Angiosarcoma | Wang L, Lao IW, Yu L, Wang J. Clinicopathological features and prognostic factors in angiosarcoma: A retrospective analysis of 200 patients from a single Chinese medical institute. *Oncol Lett*. 2017;14(5):5370–5378. |
| Papillary thyroid cancer | Maino F, Forleo R, Pacini F. Prognostic indicators for papillary thyroid carcinoma. *Expert Rev Endocrinol Metab*. 2017;12(2):101-108. |
| Uveal melanoma | Kaliki S, Shields C, Shields J. Uveal melanoma: Estimating prognosis. *Indian J Ophthalmol*. 2015;63(2):93-102. |
| Melanoma | Hsueh E, Lucci A, Qi K, Morton D. Survival of patients with melanoma of the lower extremity decreases with distance from the trunk. *Cancer*. 1999;85(2):383-388. |
| Thymoma | Gripp S, Hilgers K, Wurm R, Schmitt G. Thymoma. *Cancer*. 1998;83(8):1495-1503. |
| Follicular lymphoma | Solal-Celigny P. Follicular lymphoma international prognostic index. *Blood*. 2004;104(5):1258-1265. |
| Pancreatic adenocarcinoma | Bilici A. Prognostic factors related with survival in patients with pancreatic adenocarcinoma. *World J Gastroenterol*. 2014;20(31):10802–10812. |
| Colorectal cancer | Fleming M, Ravula S, Tatishchev SF, Wang HL. Colorectal carcinoma: Pathologic aspects. *J Gastrointest Oncol*. 2012;3(3):153–173. |
| Osteosarcoma | Lee RJ, Arshi A, Schwartz HC, Christensen RE. Characteristics and prognostic factors of osteosarcoma of the jaws: a retrospective cohort study. *JAMA Otolaryngol Head Neck Surg.* 2015;141(5):470–477. |
| Breast cancer | Cianfrocca M, Goldstein L. Prognostic and predictive factors in early-stage breast cancer. *Oncologist*. 2004;9(6):606-616. |
| Esophageal cancer | Tustumi F, Kimura CM, Takeda FR, et al.  Prognostic Factors and Survival Analysis in Esophageal Carcinoma. *Arq Bras Cir Dig*. 2016;29(3):138–141. |
| Esophageal cancer | Enzinger P, Mayer R. Esophageal cancer. *N Engl J Med*. 2003;349(23):2241-2252. |
| Duodenal adenocarcinoma | Lee H, You D, Paik K, Heo J, Choi S, Choi D. prognostic factors for primary duodenal adenocarcinoma. *World J Surg*. 2008;32(10):2246-2252. |
| Epithelioid hemangioendothelioma | Al-Qattan MM, Al-Rikabi AC. Epithelioid hemangioendothelioma of the subcutaneous tissues of a finger. *Indian J Orthop*. 2014;48(5):522–524. |
| Bladder cancer | Kucuk U, Pala EE, Cakır E, et al. Clinical, demographic and histopathological prognostic factors for urothelial carcinoma of the bladder. *Cent European J Urol*. 2015;68(1):30–36. |
| Squamous cell carcinoma of parotid | Marks M, Ryan R, Litwin M, Sonntag B. Squamous Cell Carcinoma of the Parotid Gland. *Plast Reconstr Surg*. 1987;79(4):550-554. |
| Acute lymphoblastic leukemia | Hoelzer D, Thiel E, Loffler H, et al. Prognostic factors in a multicenter study for treatment of acute lymphoblastic leukemia in adults. *Blood*. 1988;71(1):123-131. |
| Pancreatic cancer | Yamada H, Hirano S, Tanaka E, Shichinohe T, Kondo S. Surgical treatment of liver metastases from pancreatic cancer. *HPB (Oxford)*. 2006;8(2):85–88. |
| Adult acute myeloid leukemia | Grimwade D, Hills R. Independent prognostic factors for AML outcome. *Hematology*. 2009;2009(1):385-395. |
| Pheochromocytomas and paragangliomas | Nicolas M, Dahia P. Predictors of outcome in phaeochromocytomas and paragangliomas. *F1000Res*. 2017;6(2160):1-8. |
| Vaginal squamous cell cancer | Hiniker S, Roux A, Murphy J, et al. Primary squamous cell carcinoma of the vagina: Prognostic factors, treatment patterns, and outcomes. *Gynecol Oncol*. 2013;131(2):380-385. |
| Laryngeal cancer and head and neck squamous cell cancer | Mamelle G, Pampurik J, Luboinski B, Lancar R, Lusinchi A, Bosq J. Lymph node prognostic factors in head and neck squamous cell carcinomas. *American J Surg*. 1994;168(5):494-498. |
| Head and neck squamous cell cancer | Veness MJ. High-risk cutaneous squamous cell carcinoma of the head and neck. *J Biomed Biotechnol*. 2007;2007(3):80572. |
| Colon cancer | Canadian Cancer Society. Prognosis and survival for colorectal cancer. https://www.cancer.ca/en/cancer-information/cancer-type/colorectal/prognosis-and-survival/?region=on. Published 2019. |
| Squamous cell carcinoma of the skin | Cherpelis B, Marcusen C, Lang P. Prognostic factors for metastasis in squamous cell carcinoma of the skin. *Dermatol Surg*. |
| Uterine carcinosarcoma | Iwasa Y, Haga H, Konishi I, et al. Prognostic factors in uterine carcinosarcoma. *Cancer*. 1998;82(3):512-519. |
| Small intestine tumors | Chaiyasate K, Jain AK, Cheung LY, Jacobs MJ, Mittal VK. Prognostic factors in primary adenocarcinoma of the small intestine: 13-year single institution experience. *World J Surg Oncol*. 2008;6:12. |
| Mesothelioma and eye cancer | American Cancer Society, Inc., Surveillance Research. *Special Section: Rare Cancers in Adults*. American Cancer Society; 2019:30-39. https://www.cancer.org/content/dam/cancer-org/research/cancer-facts-and-statistics/annual-cancer-facts-and-figures/2017/cancer-facts-and-figures-2017-special-section-rare-cancers-in-adults.pdf. |
